# Zika Virus Infection Leads to Demyelination and Axonal Injury in Mature CNS Cultures

**DOI:** 10.3390/v13010091

**Published:** 2021-01-11

**Authors:** Verena Schultz, Stephanie L. Cumberworth, Quan Gu, Natasha Johnson, Claire L. Donald, George A. McCanney, Jennifer A. Barrie, Ana Da Silva Filipe, Christopher Linington, Hugh J. Willison, Julia M. Edgar, Susan C. Barnett, Alain Kohl

**Affiliations:** 1 Institute of Infection, Immunity and Inflammation, College of Medical Veterinary and Life Sciences, University of Glasgow, Glasgow G12 8TA, UK; verena.schultz@glasgow.ac.uk (V.S.); gmccanney@secerna.co.uk (G.A.M.); jennifer.barrie@glasgow.ac.uk (J.A.B.); chris.linington@mauter-linington.de (C.L.); hugh.willison@glasgow.ac.uk (H.J.W.); julia.edgar@glasgow.ac.uk (J.M.E.); 2 MRC-University of Glasgow Centre for Virus Research, Institute of Infection, Immunity and Inflammation, College of Medical Veterinary and Life Sciences, University of Glasgow, Glasgow G61 1QH, UK; s.l.cumberworth@gmail.com (S.L.C.); Quan.Gu@glasgow.ac.uk (Q.G.); natasha.johnson@glasgow.ac.uk (N.J.); Claire.Donald@glasgow.ac.uk (C.L.D.); Ana.daSilvaFilipe@glasgow.ac.uk (A.D.S.F.)

**Keywords:** Zika virus, mature CNS, demyelination, CCL5

## Abstract

Understanding how Zika virus (*Flaviviridae*; ZIKV) affects neural cells is paramount in comprehending pathologies associated with infection. Whilst the effects of ZIKV in neural development are well documented, impact on the adult nervous system remains obscure. Here, we investigated the effects of ZIKV infection in established mature myelinated central nervous system (CNS) cultures. Infection incurred damage to myelinated fibers, with ZIKV-positive cells appearing when myelin damage was first detected as well as axonal pathology, suggesting the latter was a consequence of oligodendroglia infection. Transcriptome analysis revealed host factors that were upregulated during ZIKV infection. One such factor, CCL5, was validated in vitro as inhibiting myelination. Transferred UV-inactivated media from infected cultures did not damage myelin and axons, suggesting that viral replication is necessary to induce the observed effects. These data show that ZIKV infection affects CNS cells even after myelination—which is critical for saltatory conduction and neuronal function—has taken place. Understanding the targets of this virus across developmental stages including the mature CNS, and the subsequent effects of infection of cell types, is necessary to understand effective time frames for therapeutic intervention.

## 1. Introduction

Zika virus (ZIKV; *Flaviviridae*) was isolated in Uganda, Africa, from non-human primates in 1947 and then mosquitoes in 1948 [1,2]. Only occasional human infections were described until its emergence on Pacific Ocean islands and later in Brazil (2015), from where the virus spread across the Americas [3,4,5,6]. *Aedes aegypti* has been recognized as a key, though not exclusive, mosquito vector for transmission to humans [7,8]. Its genome structure is comparable to that of related flaviviruses, such as dengue or yellow fever viruses [9,10]. The existence of two lineages of ZIKV, African and Asian [11], has been established, which may impact pathogenesis in humans [5], although many questions remain unanswered. The ZIKV outbreak in the Americas was caused by Asian lineage ZIKV, and an American subclade emerged during this outbreak in a susceptible population that had not been exposed to this pathogen before [12,13]. An unreported outbreak in Cuba in 2017 and import of ZIKV from Brazil to Angola suggest that surveillance activities must continue [14,15].

The outbreak in the Americas was associated with an increase in disease manifestations that were unexpected for ZIKV. Importantly, neurological complications such as Guillain–Barré syndrome (GBS), meningoencephalitis, and myelitis were reported from patients. ZIKV infections following maternal–fetal transmission (regardless of whether mothers were asymptomatic or symptomatic) were also found to result in a spectrum of congenital pathologies. These included neurodevelopmental impairments, grouped under the description “congenital Zika syndrome” (CZS), as well as fetal death. Moreover, later neurodevelopmental abnormalities have been reported, thus emphasizing the need for long-term surveillance [3,4,6,12,16,17,18,19,20,21,22]. The full spectrum of disease may indeed only become clear over time.

The neuropathological manifestations in CZS seem primarily limited to the central nervous system (CNS) [23,24]. A considerable body of work has established cellular targets of ZIKV, which encompasses many of cells linked to the development, function, and support of the CNS: neural stem and progenitor cells, neurons, neuroepithelial cells, and glial cells [25,26]. Glial cell populations are composed of several cell types, the major components of which include oligodendrocytes, astrocytes, and microglia. Oligodendrocytes play a critical role in the CNS by myelinating axons. The myelin sheath effects saltatory conduction along axons, and oligodendrocytes also provide metabolic support (e.g., glycolytic products) for axon integrity. Cross-talk between neurons and oligodendroglia is essential for proper functionality [27,28,29,30]. Damage to oligodendrocytes can lead to secondary axonal injury, resulting in morphological and molecular changes in neurons [31]. Axonal injury has been observed in a pigtail macaque model of ZIKV infection [32]. Demyelination and the infection of myelinating cells by ZIKV have been proposed as one of the mechanisms leading to fetal brain injury and neurological disease [33,34]. Importantly, the inhibition of myelination or demyelination in the CNS have been observed in human fetal ZIKV infections [35,36,37]. These findings in human infections were replicated and explored further in ZIKV animal models, which showed a significant impact of ZIKV infection on axons and myelin when infected late in gestation or shortly after birth [38,39,40,41]. Myelination in humans is a dynamic ongoing process; rapid myelination takes place across the first few years of life, but the brain may take up to 20–25 years to mature with myelination continuing post-birth [42,43]. As ZIKV infection may affect oligodendrocyte development [40], the roles of viral oligodendrocyte tropism and demyelination in ZIKV CNS pathology warrant further investigations. We have recently used a myelinating CNS neural culture system derived from *Ifnar1* knockout mice, and indeed, type I interferon deficient mice have been described to recapitulate aspects of human ZIKV infections and disease [26]. These cultures contain all major neural cell types: neurones, microglia, astrocytes, oligodendrocytes, and oligodendrocyte precursor cells [44]. Using ZIKV strain PE243, a patient-derived isolate obtained during the outbreak in Brazil, we showed that in myelinating cultures oligodendrocytes are proportionally more targeted by this virus than other cell types, affecting ongoing myelination and resulting in axonal injury [44]. ZIKV infection has been increasingly linked to CNS pathology, including myelin injury in adults: acute myelitis, encephalitis, meningoencephalitis, and encephalomyelitis [45,46,47,48,49,50,51,52,53,54,55,56]. Furthermore, ZIKV has been detected in adult human brain tissue [57]. Therefore, how and if the virus affects the mature, myelinated CNS are important questions.

Here, we investigated specifically how ZIKV PE243 affected myelin and axons in mouse-derived mature CNS cell cultures, where myelin sheaths are already established. We found that these mature cultures are vulnerable to ZIKV-induced pathology. The detection of ZIKV infection by immunofluorescence was concurrent with initial signs of myelin damage, alongside axonal injury and diminished axonal density. Transcriptomic analysis of ZIKV-infected CNS cultures showed also an upregulation of factors that have been linked to reduced myelination and demyelination. We assessed one such factor, CCL5, and showed using rat CNS culture (a model used to study myelination development) that it did indeed inhibit myelination. The transfer of UV-treated, ZIKV-inactivated supernatant to otherwise healthy CNS cultures did not result in myelin damage or axonal injury. Our data suggest a process by which ZIKV infection directly affects myelin also in the mature CNS, which may contribute to disease in adults.

## 2. Materials and Methods

### 2.1. Animals and Ethics Statement

Sprague–Dawley rats or *Ifnar1* -/- knockout mice (129S7/SvEvBrdBkl-Hprtb-m2 background, Marshall Bioresources, Hull, UK) were used for the generation of CNS myelinating cultures. All animal studies were approved by the Ethical Committee of the University of Glasgow and licensed by the UK Home Office (Project License number for *Ifnar1* knockout mice: PPL 60/4363). The extraction of genomic DNA and genotyping of mice was conducted as described previously [44].

### 2.2. Cell Culture

Reagents used for the protocols described here were obtained from Sigma-Aldrich (Gillingham, UK). Mouse CNS immature and mature myelinating cultures were generated as described earlier [58,59,60]. Briefly, E13 (E, embryonic day; number indicates days) mouse spinal cords were isolated, and the meninges were removed and enzymatically (Trypsin, 0.25 g/mL) and mechanically (Pasteur pipette) dissociated into a single cell suspension. Cells were plated at 150,000 cells per 13 mm diameter glass coverslips coated with poly-L-Lysine (MW 70,000–150,000). Cells were plated initially in 12.5% horse serum (100 µL) as a drop on the coverslips for 2 h, after which medium was topped up to 1200 µL, resulting in a ratio of 1:1 of plating medium (25% DEM (4.5 mg/mL glucose, 100 U/mL penicillin, 100 μg/mL streptomycin) 12.5% horse serum, 12.5% Hank’s buffered salt solution (with Ca^2+^/Mg^2+^)) and differentiating medium (DMEM (4.5 mg/mL glucose), 100 U/mL penicillin, 100 μg/mL streptomycin, 10 ng/mL biotin, 1% N1 medium supplement, 50 nM hydrocortisone, and 10 μg/mL insulin). Horse serum was gradually withdrawn through feeding every 2nd or 3rd day with serum-free differentiation medium, of which insulin was omitted after 12 days. Cells were maintained in 5% CO_2_ at 37 °C. 

Rat CNS myelinating cultures were generated based on our previously described methods [60,61,62]. The method is based on the generation of mouse CNS cultures with a few modifications. Briefly, the spinal cords of E15.5 embryos were dissociated, and the resulting cell suspension was plated on top of neurosphere-derived astrocytes. Neurospheres were generated from the striata of 1-day-old Sprague–Dawley rats [63] and differentiated into astrocytes as described [60]. After 5–7 days in vitro (DIV), the generated astrocytes form a monolayer onto which the spinal cord cells were added in a 50 µL drop for 2 h. Then, the media was topped up as described for the mouse cultures, and the cultures were fed in the same manner. Cells were maintained in 7% CO_2_ at 37 °C.

### 2.3. Viruses and Infection of Neural Cultures with ZIKV 

The origin, history, and preparation/growth of the low-passage Brazilian isolate of ZIKV, ZIKV/H. sapiens/Brazil/PE243/2015 (ZIKV PE243) have been previously described [44,64]. CNS cultures were infected with ZIKV at a multiplicity of infection (MOI) of 0.3 for 1 h at 37 °C in PBS supplemented with 2% fetal bovine serum (FBS). Controls (mock-infected) were treated in parallel with vehicle only (2% FBS in PBS). Following incubation, virus was aspirated, and the cultures returned to serum-free differentiation medium. Medium was replenished every 2–3 days. 

### 2.4. RNA Isolation and Purification

For RNA harvesting, the supernatant of infected cultures was removed, and cells were lysed in Trizol (Thermo Fisher Scientific, Waltham, MA, USA) for 5–10 min at room temperature; lysate was stored at −80 °C until required. For the analysis of receptor expression, we purified the RNA from 3 coverslips per *n* of immature (DIV 18 ± 1) or mature (DIV 28) CNS cultures using the PureLink^TM^ RNA Mini Kit (Thermo Fisher Scientific, Waltham, MA, USA) according to the manufacturer’s instructions with an on-column DNA digestion step (PureLink^TM^ DNase set, Thermo Fisher Scientific, Waltham, MA, USA). For RNA sequencing, we purified the RNA from 6 coverslips per *n* of mock- or ZIKV-infected CNS cultures (2 days post-infection (dpi) and 4 dpi) using the RNeasy Minikit (Qiagen, Hilden, Germany) according to the manufacturer’s instructions with an on-column DNA digestion step (DNase I, Qiagen, Hilden, Germany). 

### 2.5. RT-PCR for Receptor Expression Detection

**Figure A1:**
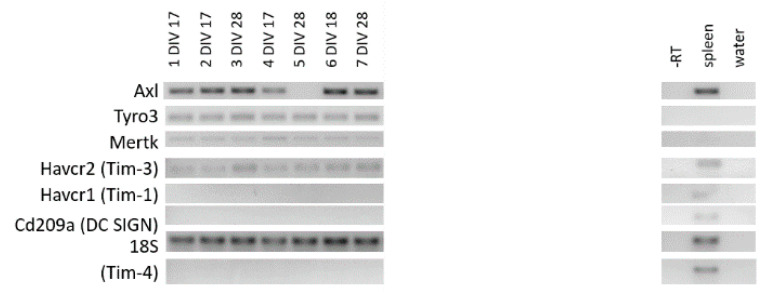
Representative image of analysis for receptor expression. Endpoint PCR products of cDNA from immature (DIV 18 ± 1) and mature (DIV 28) cultures are visualized by ethidium bromide after agarose gel electrophoreses. 18S is used as a reference gene. Controls: -RT did not contain the reverse transcriptase during cDNA generation; spleen included as reference for receptors known to be expressed by adaptive immune cells; water did not contain cDNA.

RNA samples (160 ng) were first transcribed into cDNA according to manufacturer’s instructions (QuantiTect Reverse Transcription Kit; Qiagen, Hilden, Germany) and then used for endpoint PCR (RedTaq^®^ Ready Mix^TM^ PCR reaction mix; Sigma Aldrich, Gillingham, UK). PCR was performed as follows: each reaction contained 12.5 µL RedTaq^®^ Ready Mix^TM^ (Sigma-Aldrich, Gillingham, UK), 2 µM primer (Integrated DNA technologies, Coralville, IA, USA), and 2 µL cDNA (16 ng). An initial heating step of 95 °C for 2 min was followed by 30 cycles of 95 °C for 1 min, 60 °C (18 S) or 56 °C (Axl, Mer, Tyro3 and Tim3) for 1 min, and 72 °C for 2 min. For the completion of syntheses, a final step of 72 °C for 5 min was run, and samples were cooled down to 4 °C. Primers used were previously published (Axl, Tyro3, Mertk, Tim-3, 18 S) [65,66,67]. Other primers used (Tim-1, Tim-4, DC SIGN) were designed for this study (all sequences are listed in Appendix A: Table A1). Products were analyzed by 1.3% agarose gel electrophoreses according to standard protocols. The resulting bands were visualized with ethidium bromide and gels were photographed. A summary of the results and representative gel photographs are shown in Appendix A: Table A2 and Figure A1. 

### 2.6. RNA Sequencing and Data Analysis

RNA concentration was measured with a Qubit Fluorimeter (Thermo Fisher Scientific, Waltham, MA, USA) and the RNA integrity was determined using an Agilent 4200 TapeStation (Agilent Technologies, Santa Clara, CA, USA). Samples had an average RIN of 9.3. Then, 500 ng of total RNA from each sample was used to prepare libraries for sequencing, using an Illumina TruSeq Stranded mRNA HT kit (Illumina, Cambridge, UK), according to the manufacturer’s instructions. Briefly, polyadenylated RNA molecules were captured, followed by fragmentation. RNA fragments were reverse transcribed and converted to dsDNA, end repaired, A-tailed, ligated to indexed adaptors, and PCR amplified. Libraries were pooled in equimolar concentrations and sequenced in 2 batches on an Illumina NextSeq 500 sequencer (Illumina, Cambridge, UK) using a high output cartridge, generating single reads with a length of 75 bp. At least 93.9% of the reads generated presented a Q score of 30 or above.

Prior to performing bioinformatics analysis, RNA-Seq reads quality was assessed using FastQC software (http://www.bioinformatics.babraham.ac.uk/projects/fastqc). Sequence adaptors were removed using TrimGalore (https://www.bioinformatics.babraham.ac.uk/projects/trim_galore/). After that, RNA-Seq reads were analyzed. Sequence reads were aligned to the *Mus musculus* genome (GRCm38) and downloaded via Ensembl using HISAT2. HISAT2 is a fast and sensitive splice aware mapper that aligns RNA sequencing reads to mammalian-sized genomes using an FM index strategy [68]. After the alignment, FeatureCounts [69] was used to count the reads mapping to the reference genome. The edgeR package was used to calculate the gene expression level and to analyze differentially expressed genes [70]. 

### 2.7. CCL5 Treatment during Culture Development

Rat CNS myelinating cultures were treated with CCL5 (R&D Systems, Abingdon, UK; human recombinant CCL5 (RANTES), P13501) starting on DIV 16 until DIV 24. CCL5 was reconstituted according to the manufacturer’s instructions (100 µg/mL, 0.1% BSA in PBS). The CCL5 solvent was used as vehicle control, referred to as control in figures. The final concentration of CCL5 in the culture medium following treatment was 100 ng/mL medium. At DIV 24, cultures were fixed with 4% paraformaldehyde in PBS for 20 min and either processed immediately for immunofluorescence analysis or stored at 4 °C until required.

### 2.8. Supernatant Transfer

Mouse CNS myelinating cultures were mock- or ZIKV-infected at DIV 18 as described above, and the culture supernatant was harvested at 6 dpi. Infectious virus particles were inactivated by exposure to UV-C light (8W; 254 nm at a distance of 2 cm for 4 min, with shaking after 2 min) [71]. UV-treated medium was mixed with fresh media (3:1) to prevent starving of cultures when added. Then, the mixture was applied to another set of mouse CNS myelinating cultures at DIV 18, fed every 2 days with a 1:1 mixture (UV treated media:fresh media), and fixed 6 days post-transfer (dpt) as described for ZIKV-infected cultures, and analyzed by immunofluorescence (IF) afterwards. 

### 2.9. IF Analysis of CNS Cultures

For IF analysis, mock- or ZIKV-infected cultures were fixed at the indicated times post-infection (2, 4, or 6 days) with 8% paraformaldehyde for 1 h at room temperature and subsequently stored in PBS at 4 °C. One *n* is defined as an independent, biological replicate as a set of cultures derived from the spinal cords of embryos from one pregnant dam. Per staining and/or condition 2–3 coverslips with cells were used. Post-fixation, mock- or ZIKV-infected mouse CNS myelinating cultures were permeabilized in ethanol (−20 °C; 10 min) and incubated in primary antibodies in 10% goat serum (in PBS) overnight at 4 °C. Mouse anti-ZIKV E (envelope) protein (clone 0302156; Aalto Bio, Dublin, Ireland; 1 in 500) was used in combination with one or other of the following cell-type specific antibodies: rat anti-PLP (proteolipid protein) (clone AA3 [72], kindly provided by Dr. Steven Pfeiffer, Connecticut; 1 in 400); rabbit anti-NeuN (Millipore, Darmstadt, Germany; 1 in 750), NG2 (Millipore, Darmstadt, Germany; 1 in 200). To label axons together with myelin, rat anti-MBP (myelin basic protein) (AbD Serotec, Kidlington, UK; 1 in 500) or rat anti PLP (same as above) was used in combination with mouse SMI31 anti-phosphorylated heavy and medium chain neurofilament (NF; Biolegend, San Diego, CA, USA; 1 in 1500). A further marker used for axons was mouse anti-β-Tubulin III (Sigma-Aldrich, Gillingham, UK; 1 in 200). To visualize mature neuronal cell bodies and axons, rabbit anti-NeuN and mouse SMI31 anti-phosphorylated heavy and medium chain NF were used. After washing, secondary antibodies (goat anti-mouse IgG1 or goat anti-rabbit IgG or goat anti-rat IgG Alexa 488 and goat anti-rat IgG or goat anti-mouse IgG1 or goat anti-mouse IgG2a Alexa 594; all 1 in 1000; Thermo Fisher Scientific, Waltham, MA, USA) were applied for 1 h at room temperature. Coverslips were mounted on glass slides in either Citifluor mounting medium with DAPI (1 ng/mL; Electron Microscopy Sciences, Hatfield, PA, USA) and sealed with nail enamel or Mowiol mounting medium (4.2% glycerol (*w*/*v*), 0.4% Mowiol 4–88 (*w*/*v*) (Calbiochem, San Diego, CA, USA), 2.1% 0.2 M Tris pH 8.5 (*v*/*v*)) with DAPI (1 ng/mL). 

For rat CNS cultures post-fixation, CCL5 treated or control cells were permeabilized with 0.2% Triton X-100 (Sigma-Aldrich, Gillingham, UK) at room temperature for 15 min, and unspecific binding sites were blocked with 0.2% porcine gelatin (Sigma-Aldrich, Gillingham, UK) in PBS. Primary antibodies were applied for 1 h at room temperature. To label axons and myelin, cultures were incubated with rat anti-PLP (Hybridoma derived [72]; 1 in 100) and mouse SMI31 anti-phosphorylated heavy and medium chain neurofilament (NF; Biolegend, San Diego, CA, USA; 1 in 1500). After washing, cultures were incubated with goat anti-rat IgG Alexa 488 and goat anti-mouse IgG1 Alexa 568 (1:500, Thermo Fisher Scientific, Waltham, MA, USA) for 45 min at room temperature and mounted in Vectashield (Vector Laboratories, Peterborough, UK). 

### 2.10. Image Capture and Analysis

Representative images and images for quantification were captured either by using an Olympus IX70 microscope (Olympus, Hamburg, Germany) with standard epifluorescence optics and Image Pro Plus 6 software (Media Cybernetics, Rockville, MD, USA) or by using an Olympus BX51 fluorescence microscope and Ocular software (QImaging, Teledyne Photometrics, Birmingham, UK), or on an Evos FL microscope (Thermo Fisher Scientific, Waltham, MA, USA) with integrated software. All conditions of a culture set, and each *n* per experiment, were imaged on the same microscope. To avoid any bias, areas (field of view) were either selected in the DAPI channel (blue) (mock- or ZIKV-infected cultures), or the experimenter was blinded to culture treatment before image capture (CCL5-treated cultures and controls). Then, images in all three channels were captured (blue/red/green). For quantification, 10 images were captured per coverslip using either ×20 magnification for quantification of cells and myelin in mock and ZIKV-infected cultures or ×10 for quantification of myelin and axons in CCL5-treated cultures and controls. 

For the quantification of cells, rectangular areas of interest (AOI) of 148,427 µm^2^ (cell marker and ZIKV E) and 20,000 µm^2^ (DAPI) were placed on each image, and cells within the area and cells touching northern and western borders were quantified, with cells only deemed qualified when having a DAPI +ve nucleus. The counts per AOI were converted to cells/mm^2^ by using the following formula: cell density per area of interest/area µm^2^ × 1,000,000. 

Myelin (MBP) and axons (SMI31-neurofilament) were quantified by using the Cellprofiler software [73], and the pipelines are available at https://github.com/muecs/cp. Pyknotic nuclei are distinguished from healthy nuclei by size, homogeneity, as well as DAPI staining intensity (pyknotic nuclei are condensed and intensely labelled).

### 2.11. Statistics

Analyses were performed by using GraphPad Prism 8 software (GraphPad software Inc., San Diego, CA, USA). A paired, two tailed Student’s *t* test was used to compare two groups with significance (*p* value) indicated as 0.001 to 0.01 (**). For both mock- and ZIKV-infected cultures *n* = 4, i.e., 4 independent cultures from 4 pregnant dams, were analyzed with 20–30 images per condition, i.e., 10 images per coverslip (Figure 2D). For the CCL5 treatment, *n* = 7, with 30 images per condition, i.e., 10 images per coverslip. Average values for each *n* were obtained over all images per condition.

## 3. Results

### 3.1. Myelinated, Mature CNS Cultures Are Vulnerable to ZIKV Infection

**Figure 1:**
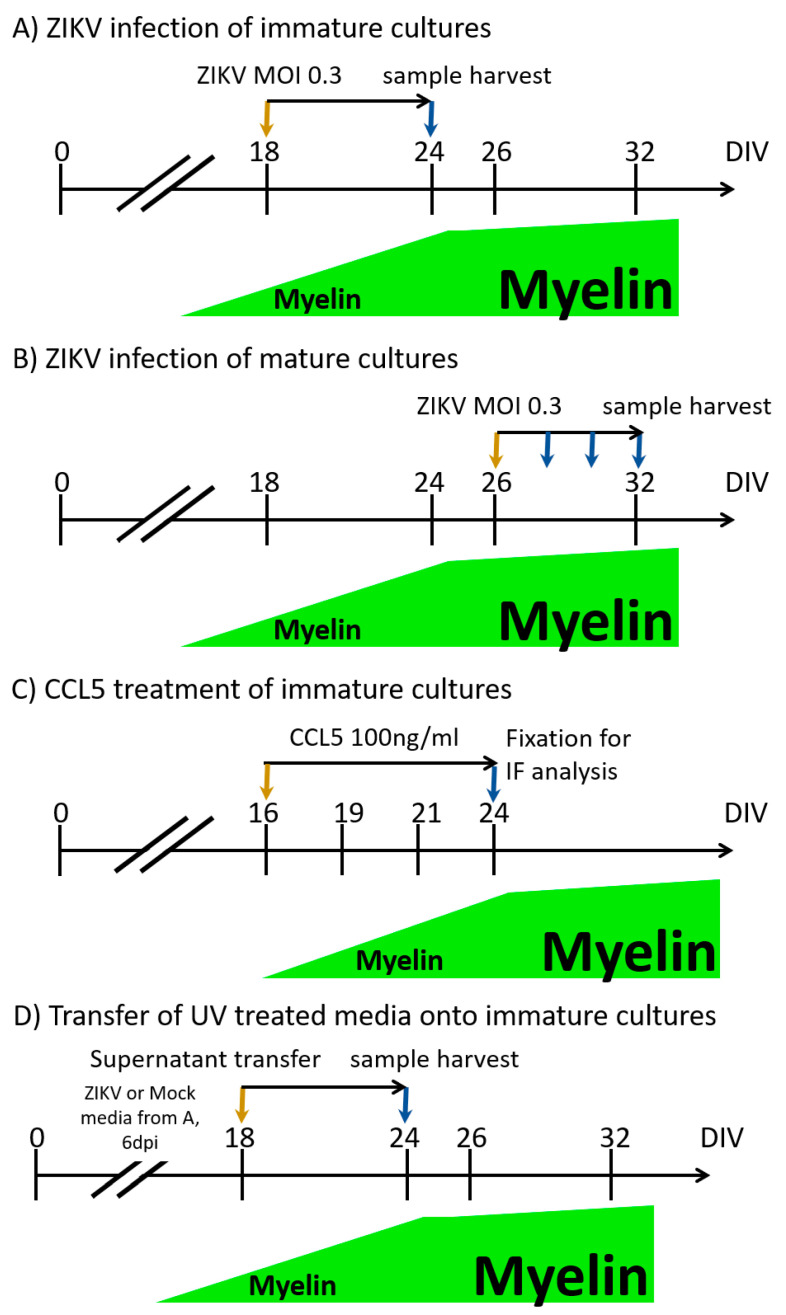
Schematic of central nervous system (CNS) cultures infected with Zika virus (ZIKV) or treated with CCL5. Orange arrows indicate the start of infection or treatment, while blue arrows indicate the day of sampling for either analysis by immunofluorescence (IF) or RNA isolation. To establish how the ZIKV infection of immature mouse CNS cultures (**A**) compares to ZIKV infection of mature CNS mouse cultures (**B**) with regard to infection and pathology, cultures were infected with ZIKV at an multiplicity of infection (MOI) of 0.3 or mock-infected (referred to as Mock in figures) and analyzed by IF after 6 dpi using our previously established protocols [44]. Cultures were always matched, so immature and mature cultures were derived from the same pool of spinal cord cells (embryonic spinal cords of one dam, taken at E13). To assess the progress of infection in mature mouse CNS cultures (**B**) and transcriptome changes, cultures were infected in the same manner, and samples were harvested at 2 and 4 dpi. (**C**) CCL5 treatment of rat CNS cultures was carried out from day in vitro (DIV) 16 until DIV 24. Cultures were fixed and analyzed by IF. (**D**) To determine whether soluble factors contributed to the observed pathology in ZIKV-infected cultures, the supernatant of ZIKV infected cultures was harvested at 6 days post-infection (dpi) (from cultures as described under (**A**)) and UV-inactivated. Then, supernatant (diluted 3:1 with fresh medium) was transferred onto immature CNS cultures, and cells were fixed 6 days post-transfer (dpt).

For this study, we used well-characterized, murine embryonic spinal cord cell-derived, CNS myelinating cultures [44,58,59]. These cultures contain the major cell types of the CNS (neurones, microglia, astrocytes, oligodendrocytes and oligodendrocyte progenitor cells (OPCs)) and form a dense network of axons in the first two weeks in vitro, becoming myelinated between day in vitro (DIV) 18 and DIV 24. Myelin within such cultures is compacted and contains normally organized nodes of Ranvier flanked by paranodes [59,60]. Usually by DIV 24, 10–15% of axons are myelinated, as assessed by IF, after which myelination reaches a plateau [59]. Mouse cultures used in this study were generated from *Ifnar1* knockout mice, as described previously [44]. These mice have an impaired type I interferon response, allowing infection in this experimental system. Cultures were distinguished between immature (DIV 18 ± 2), i.e., cultures that are not fully myelinated yet, and mature (DIV 24 and older), i.e., cultures with myelinated axons. To assess whether the pathology caused by ZIKV infection in CNS cultures occurred when myelin sheaths were already established, we carried out a comparative analysis of ZIKV infection of immature (not yet myelinated) and mature (myelinated) CNS cultures (experimental timelines described in Figure 1A,B). A single biological replicate (*n*) was defined as a culture derived by pooling all embryonic spinal cords from one pregnant dam. The cell suspension of pooled spinal cords was divided onto coverslips. CNS cultures were mock-infected (referred to as Mock in figures) or ZIKV-infected on DIV 18 ± 2 or DIV 26 ± 2 at a MOI of 0.3, until 6 dpi. We compared immature and mature cultures originating from the same biological replicate to allow direct comparisons. 

**Figure 2:**
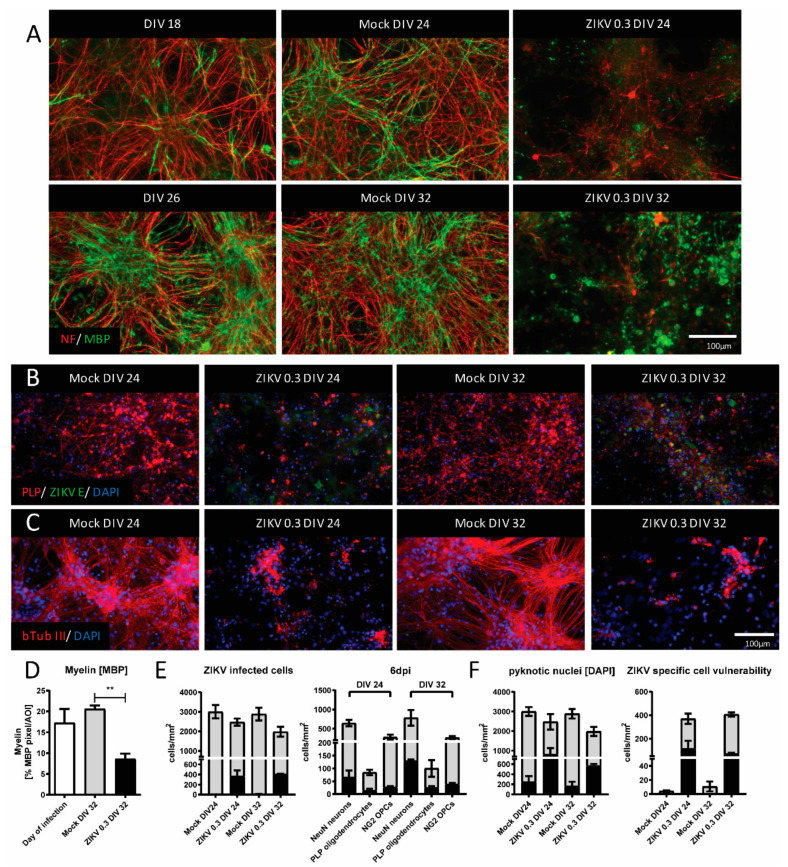
ZIKV infection causes damage to mature, myelinated CNS cultures. (**A**) Mouse CNS myelinating cultures were either mock- or ZIKV-infected (MOI 0.3) at DIV 18 (upper panel) or DIV 26 (lower panel) and analyzed by IF at 6 dpi. Mature myelin basic protein (MBP, green signal) and axon (NF, red signal) staining are shown, representative of four biological replicates (*n* = 4). (**B,C**) IF analysis of myelin (PLP, red signal) and axons (β-Tubulin III, red signal). ZIKV infection was assessed with an antibody recognising ZIKV E protein (green signal). A total of four biological replicates (*n* = 4) were analyzed. (**D**) Quantification of myelin with Cell Profiler software. *n* = 4; Mean ± SEM; paired t test; ** *p* < 0.01. (**E**) CNS cells in mature and immature cultures were quantified (*n* = 2; Mean ± SEM), to show susceptibility to ZIKV infection. Gray bars represent the overall cell number and black bars represent the number of infected cells. Left panel, overall numbers; right panel, cell types. (**F**) Cell death was quantified by assessing DAPI stained nuclei (left panel). Gray bars represent the overall density of nuclei, while black bars represent the proportion of pyknotic nuclei (*n* = 2; Mean ± SEM). A high proportion of ZIKV-infected cells underwent cell death as assessed by DAPI-stained nuclei (right panel); gray bars represent the overall density of ZIKV-infected cells, black bars represent the proportion of ZIKV-infected cells with pyknotic nuclei (*n* = 2; Mean ± SEM). ZIKV E staining showed very low background in mock-infected cultures, which are indicated here for completeness.

Initial analysis by IF with markers for axons (NF) and myelin (MBP) showed pathological effects of ZIKV in both cultures, with a significant loss of myelin observed in mature cultures (Figure 2A,D). These findings were confirmed by using additional markers for myelin (PLP) and axons (β-Tubulin III), highlighting that the overall structure of axons and myelin was pathologically altered by the infection (Figure 2B,C). From the *n* = 4 analyzed to assess the damage of myelin (Figure 2A,D), we verified the infected cell numbers and types of *n* = 2. 

The quantification of ZIKV-infected cells, specifically neurons, oligodendrocytes, and OPCs suggested similar infection patterns between the two groups, mature and immature (Figure 2E). This initial analysis included cells with intact DAPI-stained nucleus and cells with pyknotic nucleus, i.e., dying cells. When differentiated between those, pronounced cell death was observed in ZIKV-infected mature and immature cultures compared to mock controls (Figure 2F, left panel). An important number of ZIKV-infected cells were observed with pyknotic nuclei (Figure 2F, right panel). The expression of putative ZIKV receptors (Axl, Mer, Tyro3, Tim-3) [74] suggested that both immature and mature cultures express molecules required for virus binding and entry (Appendix A: Table A2 and Figure A1). Some variability in expression patterns may be due to the primary nature of the cell cultures. These findings suggested that CNS white matter structures, myelin and axons, are also vulnerable during later stages of development.

### 3.2. Timing of Myelin Damage and Axonal Injury Following ZIKV Infection

**Figure 3:**
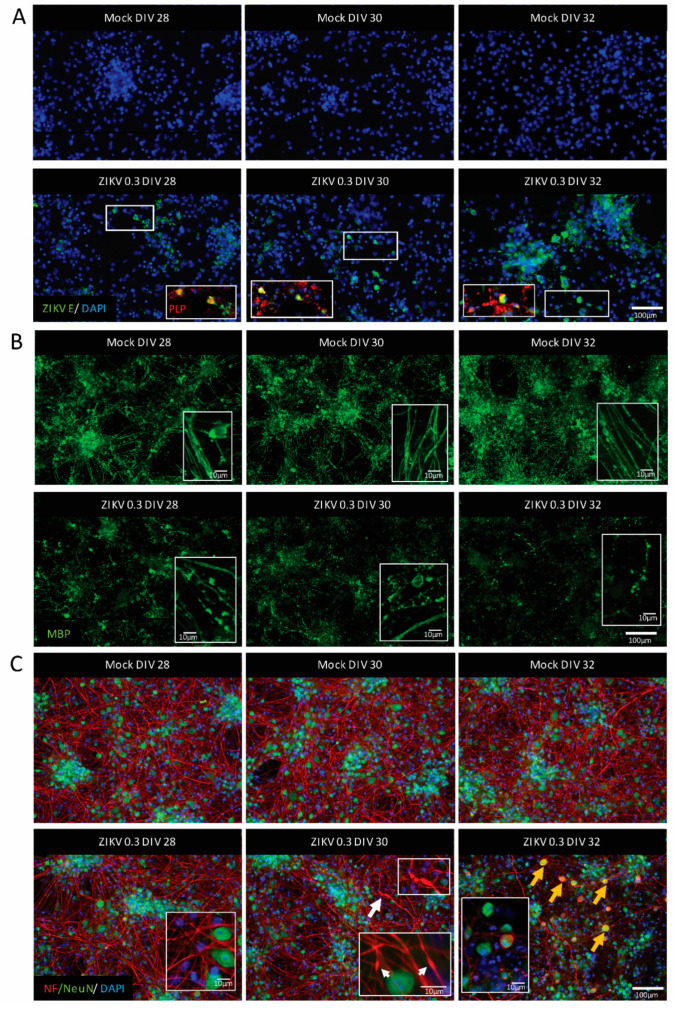
ZIKV infection of mature CNS cultures was accompanied by myelin damage and axonal injury. Representative images of mature mouse CNS cultures infected with ZIKV on DIV 26 with MOI 0.3 (*n* = 3). Images taken at 2 dpi (DIV 28), 4 dpi (DIV 30), and 6 dpi (DIV 32). Upper panels show mock-infected and lower panels show ZIKV-infected samples. (**A**) ZIKV-infected cells (ZIKV E staining, green signal), and oligodendrocyte staining as determined by co-labeling with PLP (red signal). Insets are enlarged areas of the presented image. White rectangles show the origin of the enlarged area. Scale bar = 100 µm. (**B**) Myelin sheaths visualized by MBP staining (green signal). Scale bar = 100 µm. Insets show high magnification images of myelin; scale bars = 10 µm. (**C**) Axonal damage was observed at 4 dpi (the white arrow highlights the damaged axon enlarged in the white rectangle); axonal density visualized by neurofilament (NF) staining (red signal). Neuronal cell bodies (NeuN staining, green signal) showing signs of injury by accumulating neurofilament (yellow arrows). Scale bar = 100 µm. Insets in images of ZIKV-infected cultures show high-magnification images of axons and neuronal cell bodies with white arrows indicating axonal injury; scale bar = 10 µm.

To shed light on the temporal relationship between ZIKV infection, myelin damage, and axonal injury, we infected mature myelinating CNS cultures at DIV 26 ± 2 (either mock-infected or ZIKV-infected at MOI 0.3) and assessed samples every other day (DIV 28 ± 2, 30 ± 2, 32 ± 2) by IF (Figure 1B). For clarity, one dpi equals a time period of 24 h. The foci of ZIKV-infected cells (mostly oligodendrocytes as determined by co-labeling with PLP) were identified from 2 dpi (DIV 28), when myelin damage (myelin staining by MBP) was also observed (Figure 3A,B). Myelin sheaths were disrupted as early as 2 dpi (see insets in mock- and ZIKV-infected cultures in Figure 3B), and this deteriorated further until 6 dpi, as previously observed in immature cultures [44]. The integrity of axons was assessed using immunolabelling for neurofilaments, which is a vital part of the axonal structure. The absence of staining for these filaments suggests that axons are degenerated. Axonal injury with axons swelling and/or transection was also observed (see insets in ZIKV-infected cultures, Figure 3C). Neurofilaments can accumulate ectopically in the neuronal cell body upon injury [75]. To corroborate our previous observations of neuronal cell bodies remaining mostly intact, while axons are lost or injured after ZIKV infection [44], we combined the neurofilament staining with IF for the transcriptional factor NeuN, which is typically found in all mature neuronal cell bodies. Axonal injury, at least as assessed by staining for phosphorylated neurofilaments, was first detected after 4 dpi, resulting in a loss of axonal density at 6 dpi (Figure 3C). Most NeuN-positive cell bodies appeared healthy during the entire time course, with some showing signs of injury in the form of accumulating neurofilaments at 6 dpi. In summary, this time course study of ZIKV infection in the mature CNS revealed that after the infection of oligodendrocytes, cultures demyelinate and exhibit axonal injury. 

### 3.3. Transcriptome Analysis of Mature, ZIKV-Infected CNS Cultures

**Figure 4:**
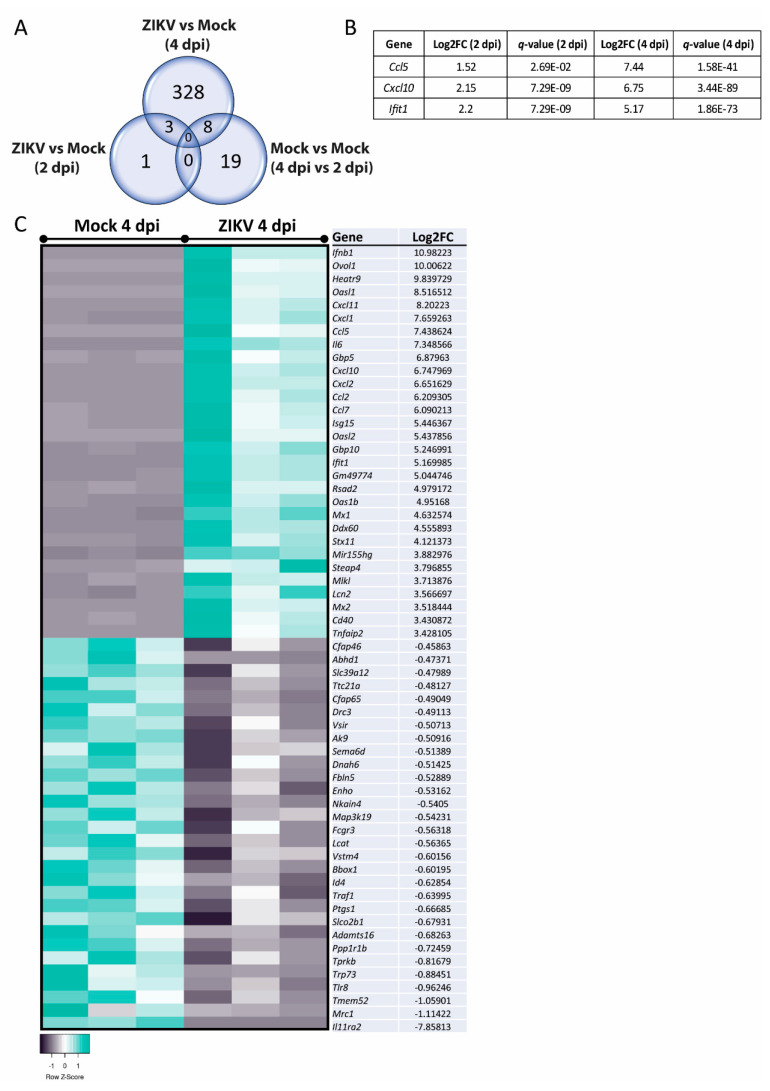
*Ccl5*, *Cxcl10*, and *Ifit1* were upregulated in mature CNS cultures at 2 dpi and 4 dpi. (**A**) Comparison of differentially expressed genes (DEGs) in ZIKV-infected versus mock-infected mature CNS cultures, at 2 dpi and 4 dpi; there were 19 DEGs unique to the dataset comparing mock-infected cultures at 4 dpi with mock-infected cultures at 2 dpi. (**B**) ZIKV-infected cultures shared three DEGs; *Ccl5*, *Cxcl10*, and *Ifit1*, with upregulation data shown in the table. (**C**) The transcriptomic analysis of independent biological replicates (*n* = 3) of ZIKV-infected mature CNS cultures at 4 dpi revealed more than 300 DEGs with a *q*-value < 0.05. The heatmap displays the top 30 up- or downregulated genes between mock- and ZIKV-infected mature CNS cultures. The heatmaps were generated with the online tool Heatmapper [77].

**Figure 5:**
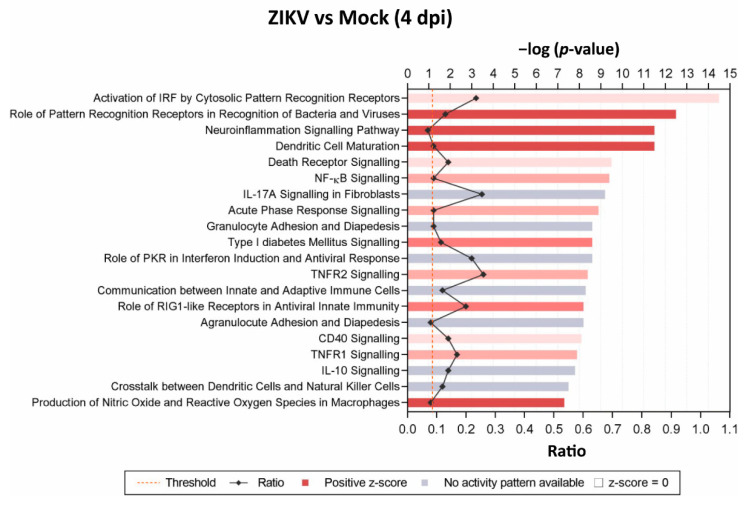
Identification of top 20 significant canonical pathways affected at 4 dpi in mature CNS cultures. Comparison of ZIKV-infected versus mock-infected mature mouse CNS culture by Ingenuity Pathway Analysis (IPA). Cellular pathways are indicated. Bars are measured against −log(*p*-values). The intensity of the color (red) correlates with z-score positivity. Positive z-scores indicate upregulated pathways, whereas negative z-scores are indicative of downregulation. Gray bars indicate that although a pathway is affected, the resulting activity (activation or repression) is unknown. The ratio reflects the number of molecules presented in the dataset that are involved in each pathway against the known total of pathway components.

To determine possible pathways and mechanisms underlying the observed myelin and axonal damage, we assessed mature, mock-infected, or ZIKV-infected cultures at 2 dpi and 4 dpi (*n* = 3 per condition) by transcriptome analysis. Differentially expressed genes (DEGs) with a false discovery rate (FDR) of <0.05 (*q*-value) were identified and revealed four DEGs at 2 dpi and 339 DEGs at 4 dpi when comparing ZIKV-infected samples versus mock-infected (Figure 4A). Comparison of the two mock-infected samples (4 dpi vs. 2 dpi) resulted in 27 DEGs, with 19 DEGs—13 upregulated and 6 downregulated DEGs at 4 dpi—unique to this dataset (Appendix B: Table A3). These genes may represent genes involved in the aging or maturation of these cultures, due to their involvement in cell cycle and cell survival. Three DEGs were shared in ZIKV-infected samples at 2 dpi and 4 dpi, *Ccl5* and *Cxcl10* (note: spelling for human proteins where used below, for clarity in capital letters), as well as the interferon stimulated gene and viral RNA sensor *Ifit1* [76] (Figure 4B). Heatmap in Figure 4C shows the top hit list of differentially regulated genes at 4 dpi. Several DEGs identified at 4 dpi were revealed by Ingenuity Pathway Analysis (IPA) as host factors involved in antiviral responses (Figure 5). Other prominent pathways upregulated at 4 dpi were linked to pathogen recognition, neuroinflammation and the production of nitric oxide (NO) and reactive oxygen species (ROS) in macrophages. The production of NO and ROS pointing toward well-known molecules in axonal injury. Additionally, TNF pathways were upregulated, confirming a cytotoxic environment and thereby our previous observation of damage in the mature CNS cultures.

**Figure 6:**
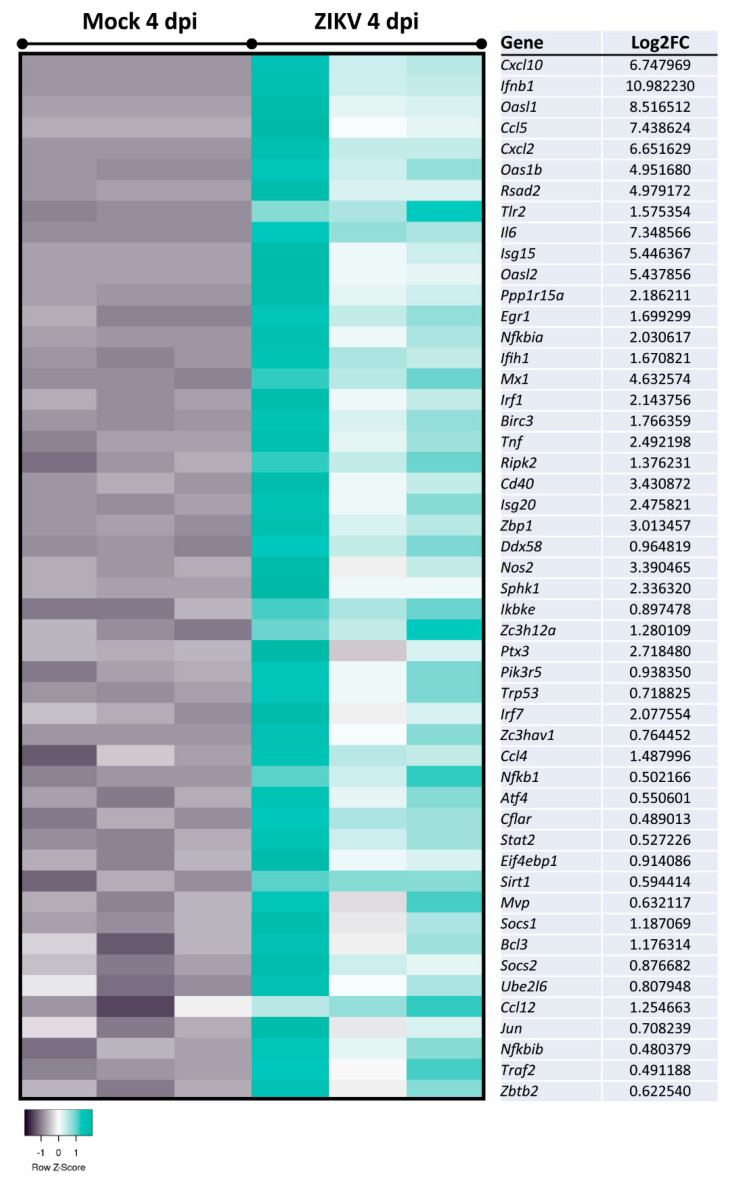
Analysis of DEGs involved in the control of viral replication in mature CNS cultures. Expression analysis of DEGs in ZIKV-infected samples versus mock-infected samples at 4 dpi by IPA revealed DEGs implicated in the reduction of viral replication, including *Ccl5*, *Cxcl10,* and *Ifit1*. Displayed DEGs were grouped and categorized using the disease and function tool. Log2FC: Log2 fold change expression value.

**Figure 7:**
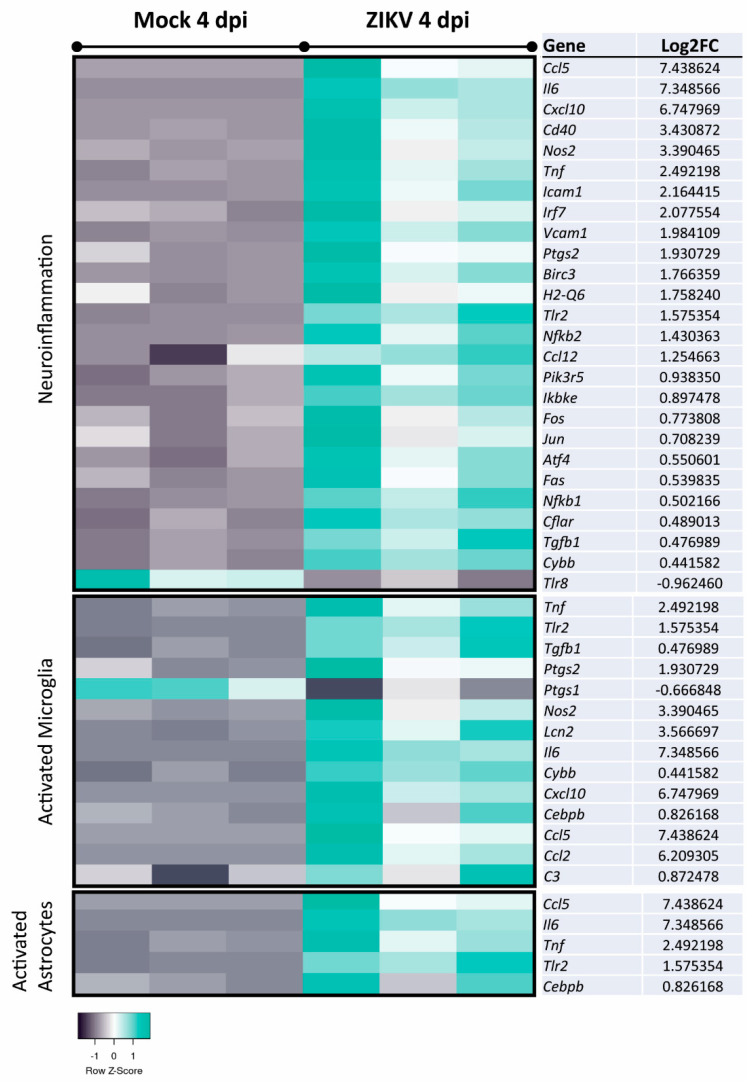
Analysis of DEGs implicated in neuroinflammatory pathways and the activation of microglia or astrocytes in mature CNS cultures. Expression analyses of DEGs in ZIKV-infected samples versus mock-infected samples at 4 dpi was performed using IPA and filtered to only include genes reported to be involved in neuroinflammation, microglia activation, or astrocyte activation (nervous system tissues, CNS cell lines, and neuroblastoma cell lines). Displayed DEGs were grouped and categorized using the canonical pathways and disease and function tool. Log2FC: Log2 fold change expression value.

**Figure 8:**
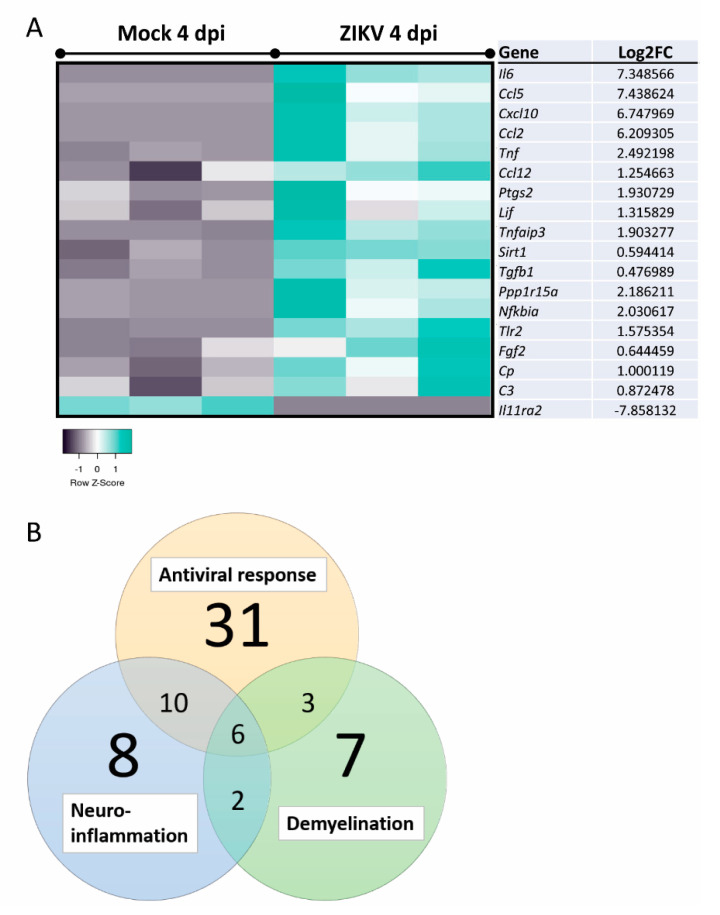
Analysis of DEGs implicated in the demyelination of axons and comparison of DEGs associated with antiviral responses, neuroinflammation, and demyelination. (**A**) Expression analyses of DEGs in ZIKV-infected samples versus mock-infected samples at 4 dpi were performed using IPA and filtered to only include genes involved in demyelination (nervous system tissues, CNS cell lines, and neuroblastoma cell lines). Displayed DEGs were grouped and categorized using the disease and function tool. Log2FC: Log2 fold change expression value. (**B**) Venn diagram of DEGs involved in antiviral response, neuroinflammation, and demyelination. Datasets were compared using VennPlex [84].

Filtering the dataset of ZIKV-infected vs. mock-infected mouse CNS cultures at 4 dpi to include only genes relevant to the control of viral replication indicated many genes involved in host responses (Figure 6). Narrowing analysis to nervous tissue and CNS cell lines by IPA identified 26 DEGs involved in neuroinflammation and 18 DEGs involved in demyelination (Figure 7 and Figure 8A). The cells responsible for inflammation in the CNS, in the absence of an adaptive immune system, are microglia and astrocytes [78,79]. We found inflammatory markers that might be associated with the activation of either cell type, but we cannot exclude other cell types as the source in this multi-cellular model (Figure 7). This further confirms the pro-inflammatory and cytotoxic environment in the mature CNS cultures after 4 dpi. The comparison of datasets—antiviral response, neuroinflammation, and demyelination—revealed that many genes were involved in these processes (Figure 8B). Thus, a pronounced antiviral and innate immune response appears to be highly involved in ZIKV-mediated damage. This analysis also showed that some DEGs (e.g., *Ccl5*, *Cxcl10*, *Tnf*, *Tgfb*, and *Il6*) are shared between these two pathways but also highlighted DEGs that are specifically associated with either neuroinflammation (e.g., *Nos2*, *Cd40*) or demyelination (e.g., *Lif, Fgf2*). *Ccl5* and *Cxcl10*, both upregulated at 2 dpi and 4 dpi, are known to support immune cell recruitment to infected tissues. In addition to their role in chemotaxis, they have previously been suggested or described to be involved in reduced myelination or demyelination, respectively [80,81]. Axonal damage at 4 dpi is likely due to neuroinflammatory processes, especially upregulation of pathways involving the production of NO and ROS, as has been previously shown [82,83].

### 3.4. CCL5 Disrupts Developmental Myelination

**Figure 9:**
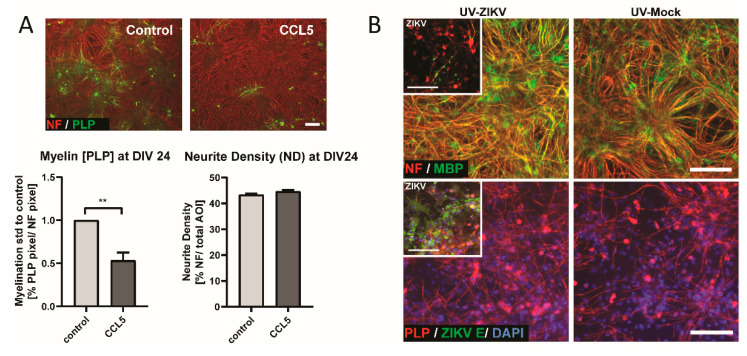
Effect of human CCL5 and UV treatment in CNS cultures. (**A**) CCL5 treatment (100 ng/mL) of rat CNS cultures from DIV 16 until DIV 24. Representative images of a control culture and a human CCL5 treated culture at DIV 24 are shown. Myelin and axons are shown by the immunofluorescence labeling of PLP (green signal) and NF (red signal). Graphs show the quantification of myelin and axons on DIV 24 per field of view of a captured image by CellProfiler software. Values for CCL5-treated cultures were normalized to control. Scale bar = 100 µm; *n* = 7; Mean ± SEM; paired *t*-test; ** *p* < 0.01. (**B**) Supernatant of mock- or ZIKV-infected immature mouse CNS cultures was collected at 6 dpi and UV-treated to inactivate viral particles. UV-treated supernatant was mixed 3:1 with fresh medium. Analysis by IF at 6 dpt onto immature cultures. Representative images of myelin staining (MBP, green signal; PLP, red signal) and axon (NF, red signal) staining at 6 dpt (DIV 24) are shown (*n* = 2). Inset images (upper panels) depict ZIKV (ZIKV E, green signal) infected CNS cells and pathology in cultures used for supernatant collection (6 dpi, DIV 24), with myelin (MBP, green signal; PLP, red signal) and neurofilament (NF, red) shown. Scale bar = 100 µm.

Due to the upregulation of *Ccl5* and *Cxcl10*, we considered their potential functions in vitro. Previously we have demonstrated that human CXCL10 has an impact on the development of myelination using rat CNS myelinating cultures [81]. Since these factors have been identified in damaged cultures and inhibit myelination, we opted to test the effect of CCL5 in a developmental, myelinating culture model [60,80]. To directly demonstrate the impact of CCL5 on myelination, we used rat CNS cultures that model developmental myelination. Cultures were treated with human CCL5 (recombinant protein) from DIV 16 until DIV 24 (see Figure 1C for illustration of the experiment), followed by IF analysis using markers for axons (NF) and myelin (PLP). We found that the addition of CCL5 led to the significant inhibition of developmental myelination, while not influencing axonal density (Figure 9A). This observation confirms that CCL5 has the potential to negatively affect myelination.

### 3.5. Transfer of UV-Treated Media from Infected Cultures Did Not Lead to Myelin and Axonal Damage in CNS Cultures

To distinguish whether ZIKV replication influenced pathology directly or by a secondary effect of ZIKV particles (even if inactivated) and/or other released cellular factors, we transferred UV- treated supernatant from two mock- or ZIKV-infected CNS cultures (6 dpi, DIV 24, mixed 3:1 with fresh media) onto two additional immature and otherwise healthy CNS cultures at DIV 18 (*n* = 2). Myelin integrity and the axonal pathology were analyzed by IF (NF, MBP, and PLP staining, respectively) at 6 dpt (DIV 24). To confirm the successful inactivation of viral particles by UV treatment, cultures subjected to supernatant transfer were also labeled with ZIKV E antibody (see Figure 1D for illustration of the experiment). CNS cultures, which received UV-treated medium from infected cultures (labeled UV-ZIKV), did not exhibit myelin or axonal damage 6 dpt, as assessed by immunolabeling for MBP, PLP, and NF and were comparable to the mock-infected cultures (labeled UV-Mock) (Figure 9B). To confirm the infectivity of the virus stock, all cultures used to generate the supernatant underwent IF analysis after the supernatant was harvested, and these cultures exhibited injury as well as high level of viral infection (Figure 9B, insert). Therefore, the demyelination and axonal damage observed in CNS cultures appears to be a result of the effects of ZIKV replication. 

## 4. Discussion

Here, we investigated the effects of a Brazilian ZIKV isolate (ZIKV PE243) on myelination, a critical process to enhance neuronal function, in the CNS. We used a mouse-embryo derived, mixed neural culture that we previously used to identify cellular targets of ZIKV infection. We observed ZIKV-associated dysmyelination within such cultures and determined that the key CNS myelinating cell type, oligodendrocytes, are predominant targets for ZIKV [44]. The causal link between GBS, a PNS neuropathy, and ZIKV infection in a predominantly demyelinating subtype of the disease is well established [12,19,45,85,86]. Although our prior studies on murine PNS myelinating cultures infected with ZIKV show poor infection of the myelin-forming Schwann cells in the PNS [44], others have shown that ZIKV infection caused cytopathic effects in Schwann cells resulting in PNS myelin degeneration [87]. The effect(s) of ZIKV in the adult, mature CNS has remained poorly understood, despite reports of acute myelitis, encephalitis, meningoencephalitis, and encephalomyelitis (neuropathologies that include myelin damage) in humans [45,46,47,48,49,50,51,52,53,54,55]. Here, we expanded our mixed neural rodent culture system to investigate the effect of ZIKV infection in mature CNS cultures, where myelination is largely complete. Importantly, we defined the temporal sequence of injury to white-matter structures in CNS cultures. These data indicate that ZIKV infection results in demyelination and axonal injury. Thus, ZIKV affects mature myelin sheaths, at least in this in vitro mouse model of infection. This is consolidated by the fact that the UV-inactivated virus has no effect on myelin integrity, suggesting that active replication is required to trigger processes leading to myelin damage. 

The mixed neural culture model we used in this study contains all major cell types of the CNS: neurones, microglia, astrocytes, oligodendrocyte precursors, and oligodendrocytes. The complexity of this model has the advantage to study viral infections and their consequences in the myelinated CNS in vitro. This model is very robust, well characterized, and has no comparable human alternatives yet. However, transcriptional changes resulting from ZIKV infection are not related or cannot be attributed to a single cell type in this complex culture model, and they must be processed by transcriptomic software to predict the contribution of the different cell types. IPA software can filter the dataset for genes known to be associated to specific processes and cell types. This analysis is based on published data and is thereby limited to only known and previously described genes and gene clusters. Single cell sequencing may be necessary for a more in-depth, detailed analysis of individual contributions. The overall response to ZIKV infection in CNS cultures included host genes typically associated with antiviral host responses such as interferon-stimulated genes [88,89]. Oligodendrocytes have been described as relatively poor sensors of viral infection, at least in the adult CNS; although antiviral response induction is limited [90], oligodendrocytes stimulate cytokine and chemokine release [91]. However, other cell types, especially microglia and astrocytes can induce antiviral responses (including following ZIKV infection) [78,79]. The exact nature of the host responses described in our mixed culture model remain to be investigated. Intriguingly, we detected the chemokines *Cxcl10* and *Ccl5* among genes upregulated by ZIKV infection. These have previously been described in arbovirus CNS following infections by other flaviviruses and alphaviruses [92]. More recently, their upregulation was shown for both ZIKV and mosquito-borne Usutu virus also in primary human astrocytes and for the latter in mouse brain [93,94]. Furthermore, both chemokines have been described to be involved in neurodegenerative processes [95,96] and have been suggested to contribute to demyelination during viral infection [97,98,99]. Through the use of myelinating CNS cultures, we have previously demonstrated that if the cultures were damaged or demyelinated, *Ccl5* was released [80]. Here, we demonstrate that the addition of human recombinant CCL5, at least in rat myelinating cultures, can inhibit myelination. The transfer of UV-treated supernatant from ZIKV- or mock-infected cultures onto separate healthy cultures did not have such an effect, which is possibly because factors act locally without release into supernatant, inactivation, or dilution. This will require further investigations. It suggests an effect of CCL5 in myelination and adding to the known functions of this chemokine not only as part of the immune response but its direct effect on neural cells. Moreover, we previously reported that the astrocyte phenotype can influence myelination. In these studies, an Illumina microarray was conducted on reactive astrocytes, and *Cxcl10* gene expression was found to be increased. To validate the effects of CXCL10 on myelination, it was added to rat developmental myelinating cultures where it significantly inhibited myelination [81]. Experiments using neutralizing antibodies to CXCL10 in myelinating cultures prevented myelination and supported its role as an inhibitor of myelination [81]. Previous work has shown that cytokines, such as Il6, Ccl7, and Ccl2, which were also among DEGs in this study, do not have an impact on developmental myelination [100]. The data shown here allows us to propose our system as a model of the mature CNS in which processes leading to demyelination could be further investigated and potential therapeutic targets assessed. Transcriptome analysis with a neuroblastoma cell line comparing Puerto Rican and Ugandan (prototype MR766) strains of ZIKV showed profound differences in how these cells responded to infection [101], and such experiments in our system may also show how African and Asian/South American strains differ in infection profiles in neural cells. 

To summarize, previous data [35,36,37] showed delayed myelination in fetal development after ZIKV infection; equivalent findings in the mature CNS are elusive. Prior to this study, it has been shown that ZIKV can affect the mature CNS by infecting cells in the adult mouse brain and triggering an inflammatory response [57]. This was accompanied by the engulfment of synapses by microglia and consequently memory impairment. Here, we showed that the virus can infect oligodendrocytes and affect myelin in a mature, myelinated CNS system. This suggests a previously undescribed pathology of ZIKV infection, which has consequences for our understanding of this virus and how it interacts with the adult CNS.

## Data Availability

Data generated or analyzed during this study are included in the published article. Raw data for quantification are available under http://researchdata.gla.ac.uk/1049/ and RNA sequencing data were deposited in The European Nucleotide Archive, Accession Number PRJEB38350.

## Appendix A

Table A1 shows the primer sequences used in this study. Figure A1 shows an example image of an endpoint PCR, with results summarised in Table A2.

**Table A1:** Primer sequences used to determine expression of putative ZIKV receptors.

Gene	Ref	Primer 1	Primer 2	Start	End	Size (bp)	Tm	Source
Axl	NM_009465.4	CCCCTGAGAACGTTAGCG	CGATCTACCATGACGTCTCGT	1244	1835	591	56	Pierce et al. 2008 [65]
Tyro3	NM_019392.2	CGATCTCCAGCTACAACGC	TAAGGCCTGAGTCGGTACG	950	1261	311	56	Pierce et al. 2008 [65]
Mertk	NM_008587.1	CTGCACAGTGAGAATCGCG	GCTTGTGTAGA CTCGGTCCG	1402	2371	969	56	Pierce et al. 2008 [65]
Havcr2 (Tim-3)	NM_134250.2	TTAGACATCAAAGCAGCCAAGG	CAGCAGAGACTCCCACTCCAAT	452	677	225	56	He et al. 2009 [66]
Havcr1 (Tim-1)	NM_001166631.1	TGCTGCTACTGCTCCTTGTG	AACCACGCTTAGAGATGCTGA	841	923	83	60	Designed for this study
Cd209a (DC-SIGN)	NM_133238.5	GAGATGACGGCTGGAATGAC	AGATGGTGGAGGGAGTTGG	710	817	108	60	Designed for this study
18S	NR_003278.1	GACTCAACACGGGAAACCTC	TAACCAGACAAATCGCTCCAC	1245	1369	124	60	Bryden et al. 2020 [67]
Timd4 (Tim-4)	NM_178759.4	GGATAGGGAATGGCACTTGA	CAACCAGACAGGACCCACA	1070	1172	103	60	Designed for this study

**Table A2:** Expression of putative ZIKV receptors in CNS myelinating cultures.

Gene	Expression at DIV 18 ± 2	Expression at DIV 26 ± 2
Axl	+	+
Tyro3	+	+
Mertk	+	+
Havcr2 (Tim-3)	+	+
Havcr1 (Tim-1)	−	−
Cd209a (DC-SIGN)	−	−
Timd4 (Tim-4)	−	−

## Appendix B

Table A3 shows DEGs which are up- or downregulated in the Mock sample at 4 dpi compared to the Mock sample at 2 dpi.

**Table A3:** The 19 DEGs unique to the comparison of Mock samples at 4 dpi vs. 2 dpi.

Gene	Description	Log2FC	*q*-Value
5430419D17Rik	RIKEN cDNA 5430419D17 gene	1.349822632	0.013727
Ptgds	Prostaglandin D2 synthase which is highly expressed in mouse brain tissue	1.159369699	6.11 × 10^−13^
Dcd dopa decarboxylase	Dcd dopa decarboxylase	0.798480908	0.001119
Prdm16os	Prdm16 opposite strand transcript	0.783089832	0.047293
C1s1	Complement component 1, subcomponent 1	0.781542798	0.032348
Sult1a1	Sulfotransferase family 1A, phenol-preferring, member 1	0.735819031	0.039275
Lyz2	Lysozyme C-2 precursor	0.67208358	0.000261
Abca9	ATP-binding cassette, sub-family A (ABC1), member 9	0.624110509	0.032074
Lrriq1	Leucine-rich repeats and IQ motif containing 1	0.548544221	0.040768
Aldh6a1	Aldehyde dehydrogenase family 6, subfamily A1	0.504553732	0.013727
Hacd4	3-hydroxyacyl-CoA dehydratase 4	0.479419852	0.040768
Prom1	Prominin 1	0.47554711	0.042655
Ccdc3	Coiled-coil domain containing 3	0.453373011	0.04515
Lims2	LIM zinc finger domain containing 2	−0.474966406	0.029954
Dbx2	Developing brain homeobox 2	−0.589232159	0.001985
Tubb6	tubulin, beta 6 class V	−0.718924133	0.028927
Cited1	Cbp/p300-interacting transactivator with Glu/Asp-rich carboxy-terminal domain 1	−0.823628338	0.032684
Kif23	Kinesin family member 23	−0.961728063	0.040768
Gm35455	Uncharacterized	−1.272445743	0.042655
